# Influence of concomitant distant lymph node metastases in metastatic hormone-sensitive prostate cancer patients with bone metastases

**DOI:** 10.1007/s00345-026-06358-5

**Published:** 2026-03-24

**Authors:** Mike Wenzel, Benedikt Lauer, Vincenzo Giuseppe Mühlthaler, Maximilian Filzmayer, Carolin Siech, Chi Le, Clara Humke, Marina Kosiba, Maximilian Kriegmair, Thomas Steuber, Felix K. H. Chun, Philipp Mandel

**Affiliations:** 1 Department of Urology, University Hospital Frankfurt, Goethe University Frankfurt am Main, Frankfurt, Germany; 2 Urologische Klinik München Planegg, Planegg, Germany; 3 Department of Urology and Urosurgery, Medical Faculty Mannheim, University Medical Center Mannheim, Heidelberg University, Mannheim, Germany; 4 Martini-Klinik Prostate Cancer Center, Department of Urology, University Hospital Hamburg-Eppendorf, Hamburg, Germany

**Keywords:** Prostate cancer, Metastatic hormone-sensitive prostate cancer, mHSPC, Metastatic burden, Survival rate, Bone metastases, Lymph node metastases

## Abstract

**Introduction:**

Most patients with metastatic hormone-sensitive prostate cancer (mHSPC) are burdened by bone metastases. However, the impact of concomitant distant lymph node metastases at diagnosis in these patients is unknown.

**Materials and methods:**

We relied on a single-center retrospective mHSPC patient cohort treated between 2014 and 2024 to compare time to metastatic castration-resistant prostate cancer (mCRPC) and overall survival (OS) between M1b vs. M1a + b mHSPC patients. Univariable and multivariable Cox regression models were applied.

**Results:**

Among 432 patients, 64% harbored M1b vs. 36% M1a + b mHSPC. Median PSA was numerically higher (82 vs. 46 ng/ml, *p* = 0.2) and rates of LATITUDE high-risk (76% vs. 55%) and CHAARTED high-volume disease (70% vs. 50%) were significantly higher in the M1a + b group (both *p* < 0.001). Regarding time to mCRPC, median time to CRPC was 26 vs. 16 months for M1b vs. M1a + b patients (hazard ratio [HR]: 1.6, *p* < 0.01). Regarding OS, median OS was not significantly different, with median OS of 53 vs. 47 months for M1b vs. M1a + b patients (*p* = 0.9). After controlling for patient and tumor characteristics in multivariable Cox regression analyses, concomitant lymph node metastases were not an independent risk for shorter time to CRPC or OS in patients with bone metastases (both *p* > 0.15).

**Conclusion:**

Patients with concomitant distant lymph node metastases in mHSPC with bone metastases (M1a + b) harbor more unfavorable baseline cancer characteristics relative to M1b without lymph node metastases, translating into significantly shorter time to mCRPC. However, after controlling for patient and tumor characteristics, neither time to mCRPC nor OS seems not be affected by concomitant lymph node metastases.

## Introduction

Prostate cancer is the second most common malignancy among men worldwide and remains a leading cause of cancer-related mortality [1, 2]. Bone metastases represent the predominant site of spread in metastatic hormone-sensitive prostate cancer (mHSPC), with 69–84% of all mHSPC patients present with bone metastases at diagnosis [3].

To better define prognosis and guide treatment strategies, mHSPC is classified based on tumor volume and tumor risk using two widely established criteria. The CHAARTED criteria differentiate between high-volume and low-volume disease and the LATITUDE criteria classify patients into high-risk and low-risk groups [4–7]. According to the CHAARTED criteria, high-volume disease is defined as the presence of visceral metastases and/or ≥ 4 bone metastases, with at least one located outside the spine or pelvis [6]. LATITUDE high-risk is classified as at least two of the following three criteria: Gleason score ≥ 8, ≥3 bone metastases, or the presence of visceral metastases [5]. Nevertheless, in these classifications the presence and extent of concomitant distant lymph nodes are not considered.

Several prognostic factors influence outcomes in mHSPC, including patient-related factors such as ECOG performance status, initial PSA level, and Gleason score [4]. The TNM classification distinguishes distant lymph node metastases (M1a) from bone metastases (M1b) regarding its prognostic effect, however both metastatic sites may occur similarly in several patients [4, 8]. Recent publications did report discrepant results concerning the oncologic outcome of patients with M1a, M1b or M1a + b [9, 10]. Additionally, current guidelines provide limited guidance on the specific management and prognostic implications of patients with distant lymph node metastases within the M1b mHSPC population, and the clinical relevance of this subgroup remains an area of ongoing discussion [4, 11].

This study aims to evaluate the additional impact of distant lymph node metastases on time to castration-resistant prostate cancer (ttCRPC) and overall survival (OS) in M1b mHSPC patients. To address this knowledge gap, we conducted the current analysis using data from the Frankfurt Metastatic Cancer Database of the Prostate (FRAMCAP) [12, 13]. We hypothesize that patients with concomitant distant lymph node metastases (M1a + b) exhibit a distinct progression and survival profile compared to patients with solely M1b mHSPC disease.

## Material and methods

### Study population

Patient data were provided by the FRAMCAP database, a single-center retrospective mHSPC patient cohort treated between 2014 and 2024, after obtaining approval from the local ethics committee (reference number: SUG-4-2024). In accordance with the principles of the Declaration of Helsinki, we conducted the current retrospective analysis of metastatic prostate cancer patients treated at the Department of Urology, between 2014 and 2024. Patients diagnosed with synchronous or metachronous mHSPC with a M1b vs. M1a + b metastatic pattern were included in the study, whereas those with non-metastatic/metastatic castration-resistant prostate cancer (mCRPC) or unknown metastatic sites were excluded. The Based on these criteria, a total of 432 patients were enrolled and classified into two subgroups according to their metastatic pattern, as defined by the TNM classification (M1b vs. M1a + b) [8]. Staging was performed using conventional methods, including bone scintigraphy and abdominal computed tomography or prostate-specific membrane antigen positron emission tomography/computed tomography (PSMA-PET/CT). All treatment decisions were made following individuals’ case discussions in a multidisciplinary tumor board.

### Definition of mCRPC

According to the EAU guidelines, mCRPC is defined and applied as disease progression despite ongoing anti-hormonal therapy, with a serum testosterone level of < 50 ng/dl (1.7 nmol/l), along with at least one of the following criteria: biochemical progression, defined as three consecutive PSA increases of at least 50% above the PSA nadir and a PSA level > 2 ng/ml, or radiological progression, evidenced by at least two new bone metastases or the presence of a visceral lesion [4, 14].

### Statistical analysis

Descriptive statistics were used to summarize the data, with categorical variables presented as frequencies and proportions. Continuous variables were reported as medians with interquartile ranges (IQR). The Chi-square test was applied to assess differences in proportions, while the t-test and Kruskal-Wallis test were used to compare distributions of continuous variables.

Primary endpoints were time to CRPC (ttCRPC) and overall survival (OS). ttCRPC was defined as the interval from the initiation of treatment for mHSPC to the progression to mCRPC or start of subsequent treatment or death. OS was defined as time interval between initiation of mHSPC treatment and death due to any cause. Both endpoints were evaluated by using Kaplan-Meier curve analyses and log-rank tests.

For all analyses, univariable and multivariable Cox regression models were applied. The univariable and multivariable Cox regression analyses included the following variables: age at metastasis, Eastern Cooperative Oncology Group (ECOG) performance status, Gleason score, high vs. low-volume CHAARTED mHSPC, synchronous vs. metachronous mHSPC, and additionally the number of systemic therapy lines for OS and specific mHSPC treatment for ttCRPC analyses.

All statistical analyses and visualizations were conducted using the R software environment for statistical computing and graphics (version 3.4.3). A p-value < 0.05 was considered as statistically significant.

## Results

The total study population included 432 patients with a median age of 69 years (IQR 63–75 years, Table 1) at the time of diagnosis of mHSPC and a median follow-up time of 28 months. The ECOG performance score was 0 in 64% of all patients. The median PSA at the time of mHSPC diagnosis 60 ng/ml (IQR 14–280ng/ml). Regarding tumor characteristics, 83% of patients presented with synchronous mHSPC. The majority of patients (74%) had a Gleason score of 8–10. Prior local therapy, including radiotherapy or radical prostatectomy, was documented in 29% of cases. Based on the CHAARTED criteria, 57% mHSPC patients harbored high-volume disease, while 63% met LATITUDE high-risk criteria. The median number of systemic treatment lines was two (IQR 2–3).

**Table 1:** Descriptive baseline characteristics of 432 metastatic hormone-sensitive prostate cancer (mHSPC) patients stratified by metastatic pattern into M1a + b versus M1b

Characteristic	*N*	Overall *N* = 432	M1a + b *N* = 157 (36%)^a^	M1b *N* = 275 (64%)^a^	*p*-value^b^
Age at diagnosis of mHSPC (years)	421	69 (63, 75)	68 (63, 75)	70 (64, 76)	0.15
PSA at mHSPC (ng/ml)	295	60 (40, 280)	82 (21, 274)	46 (12, 319)	0.2
PSA at mCRPC (ng/ml)	152	14 (3, 48)	17 (7, 37)	12 (3, 55)	0.6
Number of systemic therapy lines	430	2 (2, 3)	2 (2, 3)	2 (2, 4)	0.2
ECOG status	354				0.5
0		225 (64%)	87 (67%)	138 (62%)	
1–2		129 (36%)	43 (33%)	86 (33%)	
Gleason Score ≥ 8	389	287 (74%)	116 (82%)	171 (69%)	< 0.01
Synchronous mHSPC	430	359 (83%)	134 (85%)	225 (82%)	0.4
Local therapy	432	124 (29%)	40 (25%)	84 (31%)	0.3
MDT	359	77 (21%)	17 (13%)	60 (26%)	0.004
High volume mHSPC	333	191 (57%)	87 (70%)	104 (50%)	< 0.001
High-risk mHSPC	340	213 (63%)	96 (76%)	117 (55%)	< 0.001
Therapy mHSPC	409				
ADT mono		169 (40.2%)	42(28.7%)	127 (48.4%)	
ARPI		162 (40%)	67 (46%)	95 (36%)	
Docetaxel		47 (11%)	21 (14%)	26 (9.9%)	
Triplet therapy		19 (4.6%)	11 (7,5%)	8 (3.1%)	
NA/Other		12 (2.9%)	6 (4.1%)	6 (2.3%)	

*ADT* androgen deprivation therapy, *CRPC* castration-resistant prostate cancer, *ECOG* Eastern Cooperative Oncology Group, *IQR* interquartile range, *MDT* metastasis directed therapy, *ARPI* Androgen receptor pathway inhibitor, *PSA* prostate specific antigen, *NA* Not available

^a^Data are presented as median (IQR) or n (%)

^b^Kruskal-Wallis rank sum test; Fisher’s exact test; Pearson’s Chi-square test

### Baseline characteristics M1a + b vs. M1b

Patients were stratified according to their metastatic pattern, with 36% (*n* = 157) classified as M1a + b vs. 64% (*n* = 275) as M1b (Table 1). Median age (67 vs. 68 years, *p* = 0.1) and ECOG performance status (33% vs. 38%, *p* = 0.5) did not significantly differ between both two groups. Conversely, median PSA at the mHSPC diagnosis was higher in the M1a + b group compared to M1b, although the result was not statistically significant (82 vs. 46 ng/ml, *p* = 0.2).

When assessing tumor characteristics, the proportion of patients with a Gleason score of 8–10 was significantly higher in the M1a + b group compared to the M1b group (82% vs. 69%, *p* < 0.01). Regarding disease stratification by CHAARTED and LATITUDE criteria, the proportion of patients with high-risk disease was significantly higher in the M1a + b group vs. M1b mHSPC patients (76% vs. 55%; *p* < 0.001). Similarly, high-volume disease was more frequently observed in M1a + b than in M1b (70% vs. 50%, *p* < 0.001). Synchronous mHSPC was balanced between M1a + b vs. M1b mHSPC patients (85% vs. 82%, *p* = 0.4).

### Time to CRPC: M1b vs. M1a + b

**Fig. 1:**
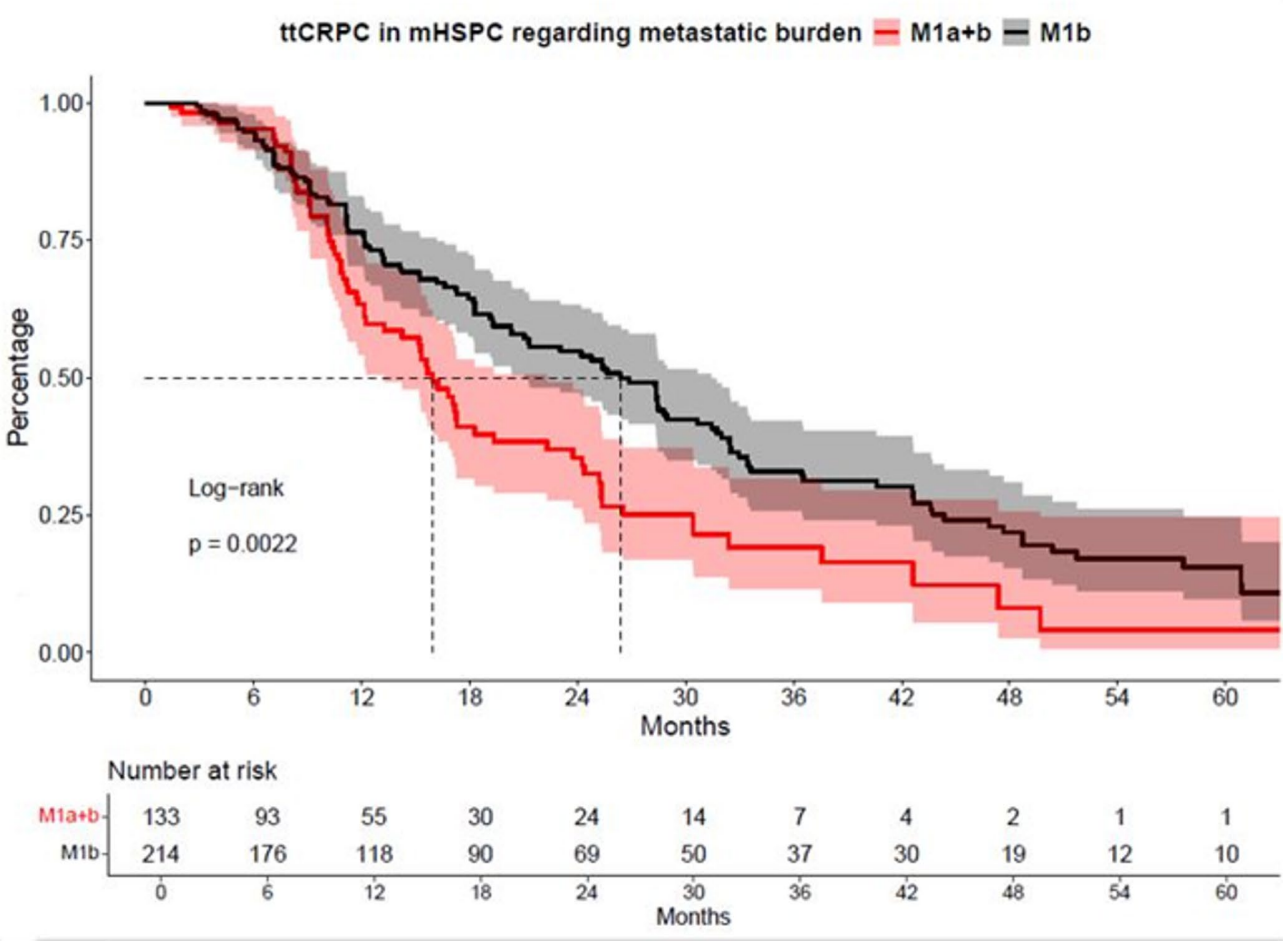
Kaplan-Meier curves depicting time to castration-resistant prostate cancer (ttCRPC) of metastatic hormone-sensitive prostate cancer (mHSPC) patients stratified according to M1a + b vs. M1b metastatic pattern

In ttCRPC analyses, significant differences between M1b vs. M1a + b group were observed (Fig. 1). Specifically, median time to CRPC was 26 vs. 16 months for M1b vs. M1a + b mHSPC patients (hazard ratio [HR]: 1.59, confidence interval (CI): 1.18–2.16, *p* < 0.01). After multivariable adjustment for baseline patient and tumor characteristics in Cox regression models, the M1a + b group showed no significant difference compared to the M1b group (HR: 1.44, CI: 0.87–2.40, *p* = 0.16, Table 2A).

**Table 2:** Univariable und multivariable Cox regression models predicting time to castration resistant prostate cancer (ttCRPC) and overall survival (OS)

	Univariable			Multivariable*		
	HR	CI	*p*-value	HR	CI	*p*-value
*ttCRPC*						
M1b	Ref	-	-	Ref	-	-
M1b vs. M1a + b	1.59	1.18–2.16	< 0.01	1.44	0.87–2.40	0.16
*OS*						
M1b	Ref	-	-	Ref	-	-
M1b vs. M1a + b	1.03	0.75–1.42	0.9	0.83	0.54–1.29	0.4

*CI* Confidence interval, *HR* Hazard Ratio

*Adjustment made for age at metastasis, ECOG performance status, Gleason score, high-volume mHSPC, de-novo mHSPC, number of therapy lines

### OS: M1b vs. M1a + b

**Fig. 2:**
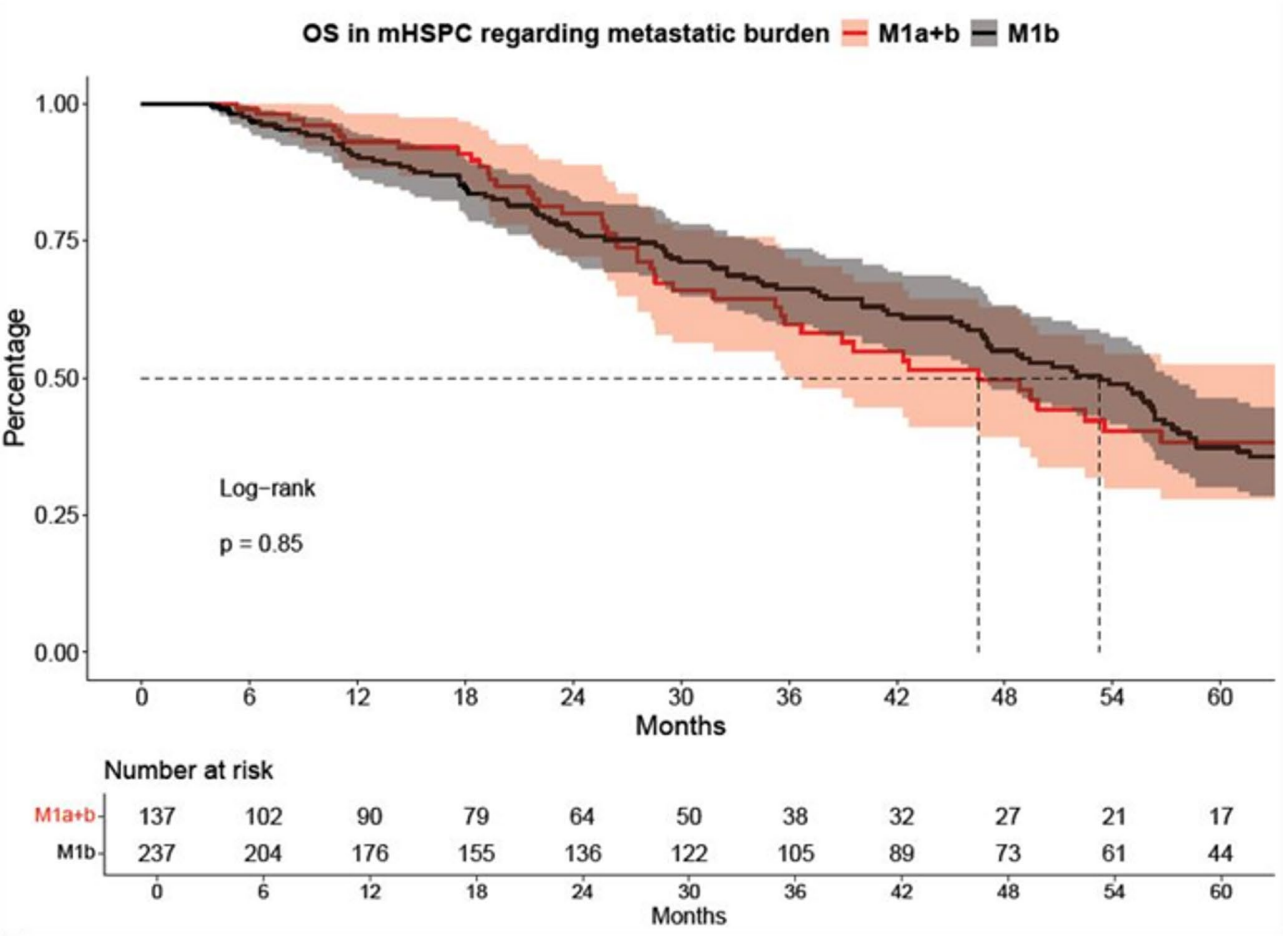
Kaplan-Meier curves depicting overall survival (OS) of hormone-sensitive prostate cancer (mHSPC) patients stratified according to M1a + b vs. M1b metastatic pattern

In OS analyses, no significant differences were observed between M1b and M1a + b patients (Fig. 2), with a median survival of 53 vs. 47 months, respectively (HR: 1.03, CI: 0.75–1.42, *p* = 0.9). Similarly, after multivariable adjustment for baseline patient and tumor characteristics in Cox regression models, analyses did not reveal any significant associations (HR: 0.83, CI: 0.54–1.29, *p* = 0.41, Table 2B).

## Discussion

We hypothesized that patients with lymph node metastases in addition to bone metastases exhibit a distinct disease trajectory and therefore differ in baseline characteristics compared to patients with bone-only metastases (M1b). We additionally initially aimed to evaluate the impact of concomitant distant lymph node metastases on ttCRPC and OS in patients with osseous M1b mHSPC. To test these hypothesizes, we relied on data from the Frankfurt Metastatic Cancer Database of the Prostate (FRAMCAP) and were able to identify several important findings.

First, we analyzed baseline and tumor characteristics in patients with mHSPC stratified by metastatic pattern M1a + b vs. M1b. Median age at diagnosis (68 vs. 70 years), and ECOG performance status showed no significant difference between the two groups (both *p* > 0.05). The PSA level in our cohort was also numerically higher (82 vs. 46 ng/ml) in the M1a + b group, but the difference did not reach statistical significance (*p* = 0.2). Compared to previously published cohorts, a study by Elewaily et al. (*n* = 129) compared patients with M1a (*n* = 28) and M1b (*n* = 91) disease. In line with our findings, the authors found no significant difference in median age (69.5 vs. 69.7 years, *p* = 0.5). Furthermore, tumor characteristics such as Gleason score and median PSA were not significantly different between the two metastatic patterns, however median difference was clinically highly relevant (M1a: 24.7 ng/ml vs. M1b: 118.8 ng/ml; both *p* > 0.10) [15]. This aligns with our findings regarding PSA, which was also higher in the M1a + b group (82 ng/ml vs. 46 ng/ml), but without reaching statistical significance, in both studies probably due to sample size limitations or wide IQRs and ranges due to number of bone metastases or concomitant metastatic lymph nodes. Differences in PSA levels between both groups may have also occurred due to the use of different imaging modalities for staging, since PSMA-PET/CT is more likely to detect low-volume lymph node metastases in patients with lower PSA levels. In contrast, Gleason score distribution differed significantly between the groups, with a higher proportion of Gleason 8–10 tumors in the M1a + b group compared to the M1b group (*p* = 0.007). Overall, our data indicate that patients with M1a + b mHSPC harbor more unfavorable baseline tumor characteristics compared to the M1b group, as evidenced by significantly higher rates of patients classified as high-risk (76% vs. 55%) and high-volume (70% vs. 50%) according to LATITUDE and CHAARTED criteria, respectively (both *p* < 0.001). These findings underline the importance of differentiating metastatic patterns when stratifying risk and selecting treatment strategies in mHSPC.

Second, we assessed cancer-control outcomes, such as ttCRPC and OS relying on Kaplan-Meier curve and log-rank test analyses. Median ttCRPC was significantly shorter in M1a + b patients compared to M1b patients (16 vs. 26 months, HR: 1.6, *p* < 0.01). In contrast, no significant difference in OS was observed between the two groups, with median survival of 47 vs. 53 months, respectively (HR: 1.03, *p* = 0.9). In multivariable analyses no significant results were found in ttCRPC and OS in comparison between the two groups. (both *p* > 0.16). In contrast to the findings of Miyake et al., who identified shorter ttCRPC as a strong negative prognostic marker for OS (*p* < 0.001), our cohort showed no significant difference in overall survival despite a shorter ttCRPC in univariable analyses. Miyake et al. demonstrated a median OS of 40.8 months in patients with ttCRPC of 0–6 months up to 70.1 months in those with a ttCRPC of more than 18.1 months. However, it is important to note that this study did not distinguish between patients with combined M1a and M1b disease and those with M1b disease alone [16]. Still, these results are in contrast with our findings, where patients with concomitant distant lymph node metastases (M1a + b) demonstrated more unfavorable baseline characteristics and a significantly shorter time to mCRPC, suggesting a more aggressive disease biology despite comparable OS.

When analyzing cancer control data in the context of the existing literature, it becomes apparent that the proportion of patients with a high tumor burden—defined by tumor volume according to CHAARTED criteria—is significantly higher in the M1a + b group. This subgroup also exhibits a significantly shorter ttCRPC, indicating a more aggressive disease course. In contrast, OS does not differ significantly between groups. This may suggest that, in the setting of progression during the mHSPC stage, OS is more strongly influenced by the choice of therapy subsequent after progression of mHSPC and the individual response to treatment. Moreover, the presence of visceral metastases may have a greater prognostic impact than lymphatic dissemination. However, the lack of significance in OS between the groups could also be attributed to the small patient number and event numbers for M1a + b patients. This hypothesis is further supported by an Asian study conducted by Wang et al., in which 870 patients with de novo mHSPC were retrospectively analyzed between 2009 and 2021. Patients were stratified according to their metastatic pattern, and OS was assessed. Those patients presenting with osseous or lymphatic metastases showed significantly better OS compared to patients with visceral metastases, particularly hepatic metastasis [17] Additionally, a study by Halabi et al. investigated the prognostic relevance of metastatic patterns in a cohort of 8,820 patients with mCRPC. Although focused on a later disease stage, the findings similarly demonstrated that patients with visceral metastases had significantly worse overall survival compared to those with lymphatic or osseous metastases [18]. To our knowledge, no study to date has specifically examined ttCRPC and OS in direct comparison between M1a + b vs. M1b metastatic patterns. Our findings therefore provide novel insights into the clinical behavior and risk stratification of mHSPC patients based on metastatic distribution.

Our study has several limitations. First, its single-center approach, which may limit the generalizability of the findings. Second, due to its retrospective design, the study is subject to inherent biases, including potential selection and information bias. Third, staging imaging was not standardized across all included patients, which may have affected the classification of metastatic patterns. Unfortunately, information regarding whether staging was performed with conventional imaging or PSMA-PET/CT was not available for all patients. As PSMA-PET/CT detects metastatic lesions with higher sensitivity, this heterogeneity may have contributed to a stage migration effect and differential classification between M1b and M1a + b disease. Although baseline characteristics were well balanced between groups, the number of patients differed substantially between the M1a + b (*n* = 157) vs. M1b (*n* = 275) cohorts, which may have reduced the statistical power to detect significant differences. Finally, data on comorbidities and PSA kinetics and exact number of lymph node and bone metastases were not available, although these factors are known to influence disease progression and OS. However, it is important to note that the evolution of systemic mHSPC therapies over the course of the observation period has also had a significant impact on oncological outcomes [19, 20].

Taken together, mHSPC patients with concomitant distant lymph node metastases in addition to bone metastases (M1a + b) present with more unfavorable baseline cancer characteristics compared to patients with solely M1b mHSPC disease. While this is associated with a significantly shorter ttCRPC in univariable analyses, ttCRPC and OS does not differ significantly between the two groups in multivariable analyses. These findings underscore the importance of considering metastatic pattern for early risk stratification, especially regarding follow-up schedules to may protect progression earlier then in M1b mHSPC patients. Future prospective multicenter studies with standardized imaging protocols and larger, balanced cohorts are warranted to validate our findings and to clarify the prognostic implications of M1b vs. M1a + b metastatic patterns in mHSPC.

## Data Availability

No datasets were generated or analysed during the current study.
